# Predictive model and determinants of under-five child mortality: evidence from the 2014 Ghana demographic and health survey

**DOI:** 10.1186/s12889-019-6390-4

**Published:** 2019-01-14

**Authors:** Justice Moses K. Aheto

**Affiliations:** Department of Biostatistics, School of Public Health, College of Health Sciences, University of Ghana, P. O. Box LG13, Legon-Accra, Ghana

**Keywords:** Child deaths, Under-five mortality, Risk factors, Predictive model, Multilevel logistic regression, Odds ratio, ROC curve, Ghana, Sub-Saharan Africa, Developing countries

## Abstract

**Background:**

Globally, millions of children aged below 5 years die every year and some of these deaths could have been prevented. Though a global problem, under-five mortality is also a major public health problem in Ghana with a rate of 60 deaths per 1000 live births. Identification of drivers of mortality among children aged below 5 years is an important problem that needs to be addressed because it could help inform health policy and intervention strategies aimed at achieving the United Nations SDG Goal 3 target 2. The aim of this study is to develop a predictive model and to identify determinants of under-five mortality.

**Method:**

The 2014 Ghana Demographic and Health Survey data was used in this study. Analyses were conducted on 5884 children. The outcome variable is child survival status (alive or dead). Single level binary logistic and multilevel logistic regression models were employed to investigate determinants of under-five mortality. The fit of the model was checked using Variance Inflation Factor and Likelihood Ratio tests. The Receiver Operating Characteristic curve was used to assess the predictive ability of the models. A *p*-value< 0.05 was used to declare statistical significance.

**Results:**

The study observed 289 (4.91%) deaths among children aged below 5 years. The study produced a good predictive model and identified increase in number of total children ever born, number of births in last 5 years, and mothers who did not intend to use contraceptive as critical risk factors that increase the odds of under-five mortality. Also, children who were born multiple and residing in certain geographical regions of Ghana is associated with increased odds of under-five mortality. Maternal education and being a female child decreased the odds of under-five mortality. No significant unobserved household-level variations in under-five mortality were found. The spatial map revealed regional differences in crude under-five mortality rate in the country.

**Conclusion:**

This study identified critical risk factors for under-five mortality and strongly highlights the need for family planning, improvement in maternal education and addressing regional disparities in child health which could help inform health policy and intervention strategies aimed at improving child survival.

**Electronic supplementary material:**

The online version of this article (10.1186/s12889-019-6390-4) contains supplementary material, which is available to authorized users.

## Background

Though there has been a significant reduction in Under-five mortality globally over the years, it is still a major public health problem in developing countries, especially in Sub-Saharan Africa where the rates have been persistently higher compared to other regions from 1990 to 2016. The global under-five mortality rate in the year 2016 was 41 deaths per 1000 live births, a decline over the previous rate of 93 deaths per 1000 live births in the year 1990. This represents 56% reduction in global under-five mortality rates [1]. However, the target is to reduce under-five mortality to at least 25 per 1000 livebirths by 2030 according to the Sustainable Development Goal (SDG) 3 target 2.1 [2]. Globally, the under-five mortality dropped significantly from 216 deaths per 1000 livebirths in 1950 to 38·9 deaths per 1000 livebirths in 2017. Disparities exist in this reduction across countries. According to estimates from the Global Burden of Disease (GBD) 2017 SDG Collaborators, many countries are on track for achieving the target of at least 25 deaths per 1000 livebirths by 2030. However, about 31 countries/territories would need to achieve annual rates of decline from 2015 to 2030 that are two to ten times higher than what was recorded for 1990–2015 in order to achieve this goal. [3]. Despite the substantial decline in global under-five mortality, the rates remain high in Sub-Saharan Africa where many countries like Ghana in the region failed to meet the Goal 4 of the Millennium Development Goals (MDGs) targets which aimed at a two-thirds reduction in the under-five mortality rate by 2015. In 2015, the under-five mortality rate in Sub-Saharan Africa was 79 deaths per 1000 live births compared to the global rate of 41 deaths per 1000 live births in the same year [1]. The under-five mortality rate in Ghana is still high with a rate of 60 deaths per 1000 live births in 2014 which fell short of the target set in the Ghana Under-five Child Health Policy 2007–2015 which targeted a reduction in under-five mortality to 40 deaths per 1000 live births by 2015 [4, 5].

In Ghana, several national policies, strategies and interventions with notable ones among them been the Child Health Policy 2007–2015, Community-based Health Planning and Services (CHPS) policy and National Health Insurance (e.g. free maternal delivery services, free treatment of children aged below 18 years) were launched to improve and promote health of Ghanaian children [4–6]. Despite all these initiatives, under-five mortality in Ghana is still high in the country. A recent study conducted in 2016 among 46 African countries reported that Ghana is among 8 countries who are making very little progress towards reduction in under-five mortality [7]. This warrant further examination of factors that may be militating against the expected reduction in the under-five mortality in the country.

There are limited studies examining risk factors associated with under-five mortality in Ghana. Majority of the few studies [8, 9] conducted to examine factors associated with under-five mortality in the country were health facility-based which are not representative of the under-five deaths in the general population of children aged below 5 years in the country. Also, there is information on under-five mortality prevalence in Ghana [4] but limited information is published on socioeconomic and environmental factors, especially modifiable risk factors that could explain the high rate of under-five mortality in the country using a nationally representative data.

The primary objective in this study is to identify critical risk factors associated with under-five mortality using a nationally representative data on children aged below 5 years and to provide a predictive model for under-five mortality. Another specific objective is to apply multilevel modelling techniques to explore unobserved household level variations in under-five child mortality while simultaneously adjusting for potential child, household and community-level risk factors. The study will also develop spatial map for crude under-five mortality rate by region. The goal is to use the findings from this study to inform and strengthen appropriate national policies and intervention strategies aimed at reducing under-five mortality in the country.

## Methods

### Study population

This study used data from the 2014 Ghana Demographic and Health Survey (GDHS) which is a nationally representative cross sectional study [4]. The data were obtained from the DHS MEASURE Program [10] which is freely available online and contain information on a wide range of population, health and nutrition indicators such as childhood mortality, maternal and child health, use of family planning methods, nutritional status of women and children as well as household socioeconomic variables. The GDHS employed a two-stage sample design to select respondents for the study. Detailed survey methods are published elsewhere [4]. Briefly, a nationally representative samples of 12,832 households from 427 clusters (primary sampling unit) comprising of 216 and 211 urban and rural centres, respectively were selected and 11,835 eligible households were interviewed. Data were collected on 9396 women of reproductive age (15–49 years) and 4388 men aged 15–59 years. Data on 5884 children aged below 5 years were generated from the interviewed women residing in 4086 households in the main survey on which the analysis in this study was based. Complete birth histories were collected including month and year of each biological child’s birth and death. These data were used to identify the number of children born in the last 5 years and child age at death. Retrospective information was obtained about children that died in the last 5 years based on information on all births to a woman within 5 years preceding the survey (i.e. from 2009 to 2014) [4].

However, the models fitted to the data were based on 5883 children residing in 4085 households from 427 clusters due to a missing observation on national health insurance status variable which is one of the important potential risk factors for health outcomes [11–13] considered in this study.

### Outcome variable

The primary outcome of interest in this study was child survival status categorised as being alive (coded as 1) or dead (coded as 0).

### Explanatory variables

The primary potential modifiable risk factors considered in this study include maternal contraceptive use and intention, maternal educational level, total children ever born, number of children under-five in household, number of births in last 5 years, household wealth status, and national health insurance status. The study also adjusts for potential confounders such as type of birth, sex of child, current age of the respondent, religion, place of residence and region of residence. Consideration of these variables were informed by the literature on child health and survival in developing countries [11–18].

### Statistical analysis

Descriptive statistics were used to summarize the distribution of selected background characteristics of children aged below 5 years. Categorical variables were summarised using frequencies with their associated percentages while continuous variables were summarised using mean with their associated standard deviation or median with their associated interquartile range if the variable violates the normality assumption. Further analyses were conducted to examine child, household and community-level factors that might be significantly associated with under-five mortality and explored unobserved household level effects on under-five mortality. Predictive models for under-five mortality were also developed. To achieve the objectives, single level and multilevel (mixed effects) binary logistic regression models were employed and model parameters were obtained using maximum likelihood. The goodness of fit for the fitted models were examined using likelihood ratio test (LRT), Akaike Information Criterion (AIC) and Bayesian Information Criterion (BIC). McFadden Pseudo R^2^ was also used to further test the fit of the preferred model. To check multicollinearity, generalised variance-inflation factor (GVIF) [19] was employed and a GVIF value below 10 was considered acceptable [20]. To examine the predictive ability of the multiple logistic regression models, we employed the Receiver Operating Characteristic (ROC) Curve. A *p*-value below 0.05 was used to declare statistical significance.

Spatial map of crude under-five mortality rate by region was produced by first aggregating the number of under-five deaths by region using the same 2014 Ghana Demographic and Health Survey dataset used in this paper as well as a regional shapefile for Ghana. All statistical analyses were done in R [21].

## Results

### Sample characteristics

Out of the 5884 children below 5 years in the data set, 289 (4.91%) of them were reported dead, 5597 were born singletons, and 283 (4.81%) were born very small in size. The proportion of children belonging to women aged 15–19 and 45–49 years are 3.52 and 2.97%, respectively. Majority of the children belonged to women with secondary education (40.94%) while 34.70% of them belonged to women with no education. About 72% of children belonged to women who were not using any form of contraceptive method at the time of the survey. In total, 4157 (70.65%) of the children belonged to household with non-piped source of drinking water while 1844 (31.34%) of them belonged to households without toilet facilities. A total of 616 (10.47%) children belonged to women who delivered through caesarean section. Majority (37.68%) of the children belonged to women who are non-users of contraceptive but intend to use it later. Majority (71.02%) of the children belonged to women with Christian religion while 3190 (54.21%) of them belonged to poor households. 1795 (30.52%) of the children were delivered at home. About 6 out of every 10 children reside in the rural communities and majority (15.33%) of the children reside in the northern region of the country (Table 1).

**Table 1:** Demographic and socioeconomic characteristics

Characteristics	n (%)
Child level factors	
Child is alive	
No	289 (4.91)
Yes	5595 (95.09)
Type of birth	
Single birth	5597 (95.12)
Multiple births	287 (4.88)
Sex of child	
Male	3066 (52.11)
Female	2818 (47.89)
Size of child at birth	
Very large	932 (15.84)
Larger than average	1989 (33.81)
Average	1944 (33.04)
Smaller than average	719 (12.22)
Very small	283 (4.81)
Don’t know	16 (0.27)
Mother/ Household level factors	
Respondent age (mean ± SD)	30.6 ± 6.89
Age in 5-year groups	
15–19	207 (3.52)
20–24	1009 (17.15)
25–29	1494 (25.39)
30–34	1409 (23.95)
35–39	1061 (18.03)
40–44	529 (8.99)
45–49	175 (2.97)
Highest education level	
No education	2042 (34.7)
Primary	1209 (20.55)
Secondary	2409 (40.94)
Higher	224 (3.81)
Religion	
Islam	1204 (20.46)
Christian	4179 (71.02)
Traditionalist/Spiritualist	237 (4.03)
No religion	264 (4.49)
Number of children < = 5 (median(IQR))	2 (1)
Total children ever born(median(IQR))	3 (3)
Births in last five years (median(IQR))	2 (1)
Sex of household head	
Male	4497 (76.43)
Female	1387 (23.57)
Current usage of contraceptive	
Using some method	1642 (27.91)
Not using any method	4242 (72.09)
Source of drinking water	
Pipe drinking water	1600 (27.19)
Non-piped drinking water	4157 (70.65)
Not dejure resident	127 (2.16)
Type of toilet facility	
Flush/pit/bucket toilet in household	3913 (66.5)
No toilet in household	1844 (31.34)
Not a dejure resident	127 (2.16)
Caesarean section delivery	
No	5268 (89.53)
Yes	616 (10.47)
Wealth index	
Rich	1611 (27.38)
Poor	3190 (54.21)
Average	1083 (18.41)
Contraceptive use and intention	
Using modern method	1462 (24.85)
Using traditional method	180 (3.06)
Non-user - intends to use later	2217 (37.68)
Does not intend to use	2025 (34.42)
Covered by health insurance	
No	1796 (30.53)
Yes	4087 (69.47)
Delivered at home	
No	4087 (69.48)
Yes	1795 (30.52)
Community level factors	
Place of residence	
Urban	2344 (39.84)
Rural	3540 (60.16)
Region	
Western	582 (9.89)
Central	603 (10.25)
Greater Accra	460 (7.82)
Volta	481 (8.17)
Eastern	545 (9.26)
Ashanti	599 (10.18)
Brong Ahafo	653 (11.1)
Northern	902 (15.33)
Upper East	551 (9.36)
Upper West	508 (8.63)

*IQR* Interquartile range, *SD* Standard deviation

### Risk factors associated with under-five mortality

To arrive at the final model, single level binary logistic regression analysis was first performed for child level factors, followed by child and maternal/household level factors and finally for child, maternal/household and community-level factors resulting in 3 models. Among the 3 models, model 3 provided a good fit to the data. A McFadden Pseudo R^2^ = 0.26, which measures the proportion of variability in the outcome explained by the risk factors indicated that the fit of the model was good. Results from models 1–3 are presented in Table 2.

**Table 2:** Risk factors for under-five mortality using multiple logistic regression models

	Model 1	Model 2	Model 3
	aOR (95% CI)	aOR (95% CI)	aOR (95% CI)
Type of birth			
Single birth	1.00 (Reference)	1.00 (Reference)	1.00 (Reference)
Multiple birth	0.22 (0.16, 0.31)***	0.48 (0.31, 0.76)**	0.47 (0.30, 0.75)**
Sex of child			
Male	1.00 (Reference)	1.00 (Reference)	1.00 (Reference)
Female	1.17 (0.92, 1.49)	1.31 (1.00, 1.72)*	1.31 (1.01, 1.72)*
Respondent’s current age	–	1.03 (0.99, 1.06)	1.03 (0.99, 1.06)
Highest education level			
No education		1.00 (Reference)	1.00 (Reference)
Primary		1.62 (1.09, 2.44)*	1.45 (0.96, 2.21)
Secondary		1.27 (0.87, 1.84)	1.22 (0.82, 1.81)
Higher		0.85 (0.41, 1.91)	0.95 (0.45, 2.15)
Religion			
Islam		1.00 (Reference)	1.00 (Reference)
Christian		1.27 (0.91, 1.77)	1.06 (0.73, 1.54)
Traditionalist/spiritualist		1.13 (0.55, 2.62)	1.13 (0.54, 2.63)
No religion		0.99 (0.53, 2.00)	0.89 (0.47, 1.83)
Number of children < 5 years	–	6.72 (5.48, 8.30)***	7.2 (5.82, 8.96)***
Wealth index			
Rich		1.00 (Reference)	1.00 (Reference)
Poor		0.95 (0.65, 1.38)	1.19 (0.72, 1.96)
Average		1.06 (0.70, 1.62)	1.15 (0.72, 1.83)
Total children ever born	–	0.81 (0.73, 0.90)***	0.81 (0.73, 0.9)***
Births in last five years	–	0.17 (0.13, 0.21)***	0.16 (0.12, 0.21)***
Contraceptive use/ intention			
Using modern method		1.00 (Reference)	1.00 (Reference)
Using traditional method		2.28 (0.68, 14.20)	2.2 (0.65, 13.78)
Non-user - intends to use later		0.73 (0.49, 1.08)	0.76 (0.51, 1.12)
Does not intend to use		0.55 (0.37, 0.81)**	0.56 (0.37, 0.82)**
Covered by health insurance			
No		1.00 (Reference)	1.00 (Reference)
Yes		0.97 (0.72, 1.30)	0.99 (0.72, 1.33)
Place of residence			
Urban			1.00 (Reference)
Rural			1.03 (0.71, 1.49)
Region			
Western			1.00 (Reference)
Central			0.43 (0.21, 0.84)*
Greater Accra			0.9 (0.38, 2.16)
Volta			0.39 (0.18, 0.83)*
Eastern			0.33 (0.16, 0.64)**
Ashanti			0.46 (0.23, 0.89)*
Brong Ahafo			0.48 (0.23, 0.96)*
Northern			0.26 (0.13, 0.50)***
Upper east			0.48 (0.22, 1.04)
Upper west			0.38 (0.18, 0.76)**

*aOR* Adjusted odds ratio, *CI* Confidence interval

*: *p*-value < 0.05. **: *p*-value < 0.01. ***: *p*-value < 0.001

Furthermore, we extend model 3 to a multilevel (mixed effect) logistic regression model (model 4) to explore any unobserved household-level effects on the under-five mortality. Presented in Additional file 1: Table S1 are the results for model 4. Comparing models 3 and 4, model 3 is preferred (Additional file 2: Table S2). Thus, no significant unobserved household-level variations were observed in under-five mortality outcome after adjusting for the risk factors in the model.

The primary modifiable risk factors significantly associated with under-five mortality were maternal contraceptive use and intention, number of children under-five in household, total children ever born and number of births in last 5 years. Though maternal educational level was significant in the child and maternal/household model (Table 2, model 2), its significance disappeared after adjusting for community level factors (i.e. place and region of residence) (Table 2, model 3). Modifiable risk factors not significantly associated with under-five mortality were household wealth status and national health insurance status. Confounding factors significantly associated with under-five mortality were type of birth, sex of child and region and those not significantly associated with under-five mortality were mother’s age, religion and place of residence.

Children from mothers who do not use and do not intend to use contraceptive has decreased odds of child survival compared to their counterparts from mothers who were using modern contraceptive method (aOR = 0.56 [95% CI: 0.37, 0.82]). Contrary to expectation, increase in number of children under-five in household is associated with increased odds of child survival (aOR = 7.2 [95% CI: 5.82, 8.96]). Total children ever born (aOR = 0.81 [95% CI: 0.73, 0.90]) and number of births in last 5 years (aOR = 0.16 [95% CI: 0.12, 0.21]) were associated with decreased odds of child survival. Children from mothers with primary education have increased odds of child survival compared to children from mothers with no education (aOR = 1.62 [95% CI: 1.09, 2.44]) (Table 2, model 2). Being a female child increased the odds of child survival compared to being a male child (aOR = 1.31 [95% CI: 1.01, 1.72]) and children born multiple has decreased odds of child survival compared to children born singletons (aOR = 0.47 [95% CI: 0.30, 0.75]).

Children residing in Central (aOR = 0.43 [95% CI: 0.21, 0.84]), Volta (aOR = 0.39 [95% CI: 0.18, 0.83]), Eastern (aOR = 0.33 [95% CI: 0.16, 0.64]), Ashanti (aOR = 0.46 [95% CI: 0.23, 0.89]), Brong Ahafo (aOR = 0.48 [95% CI: 0.23, 0.96]), Northern (aOR = 0.26 [95% CI: 0.13, 0.50]) and Upper West (aOR = 0.38 [95% CI: 0.18, 0.76]) regions have decreased odds of child survival compared to their counterparts residing in the Western region of the country (Table 2).

### Goodness of fit and predictive ability of the models

Likelihood ratio test (deviance, AIC, and BIC Values) suggests that model 3 in Table 2 provides a good fit to the data (Additional file 2: Table S2).

Examination of the GVIF values for the preferred model (model 3) reveals that all the GVIF values are less than 4 which is far less than the cut-off value of 10 [20] and the mean GVIF is 1.99 which is less than 6, suggesting no problem with multicollinearity in the model (Additional file 3: Table S3).

**Fig. 1:**
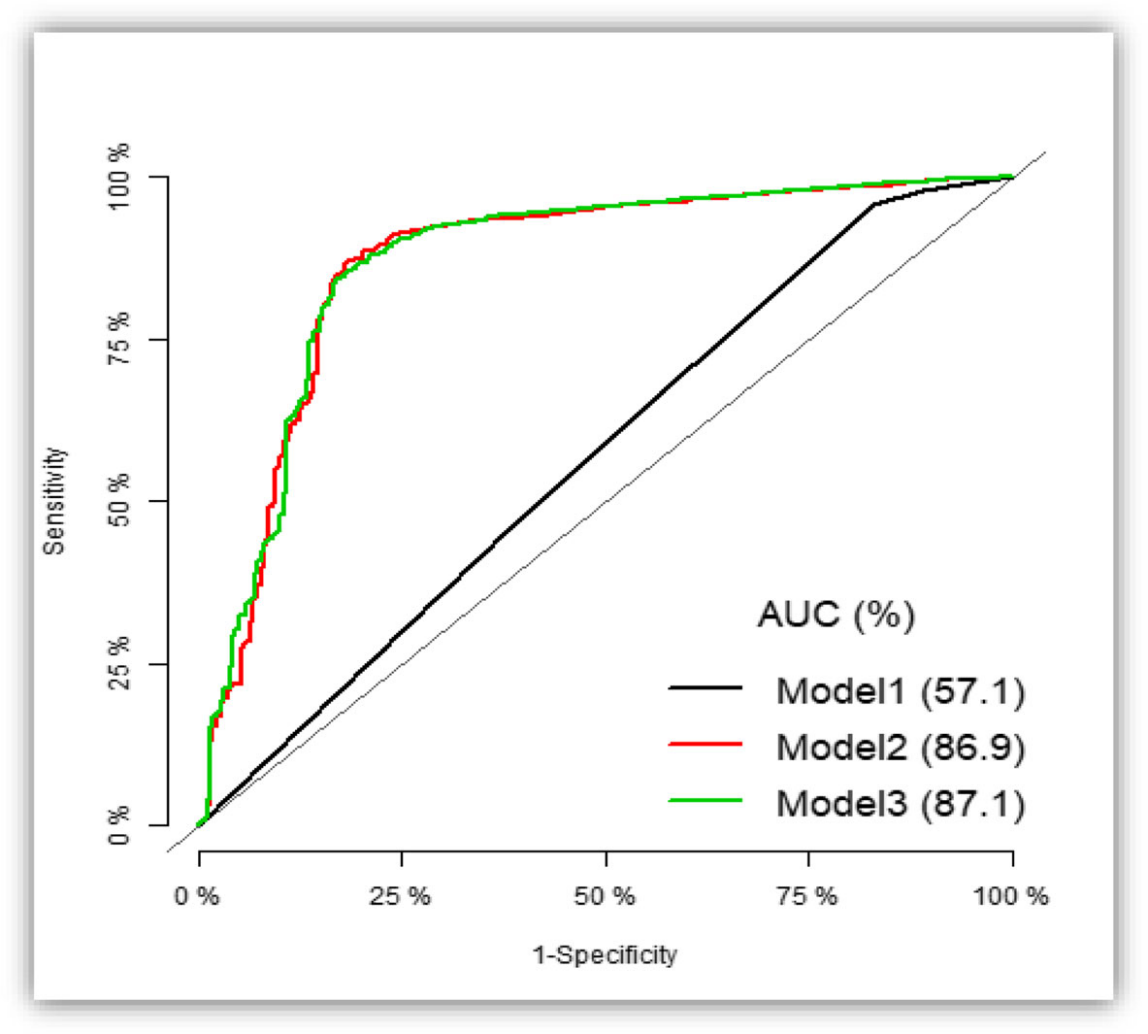
Area Under Receiver Operating Characteristic curve of multiple logistic models for predicting under-five mortality outcome

Presented in Fig. 1 is the area under the Receiver Operating Characteristic (ROC) curve. Figure 1 shows an area under the ROC curve of 87.1% for our preferred model (model 3), indicating a good predictive ability of the fitted model to predict under-five mortality.

**Fig. 2:**
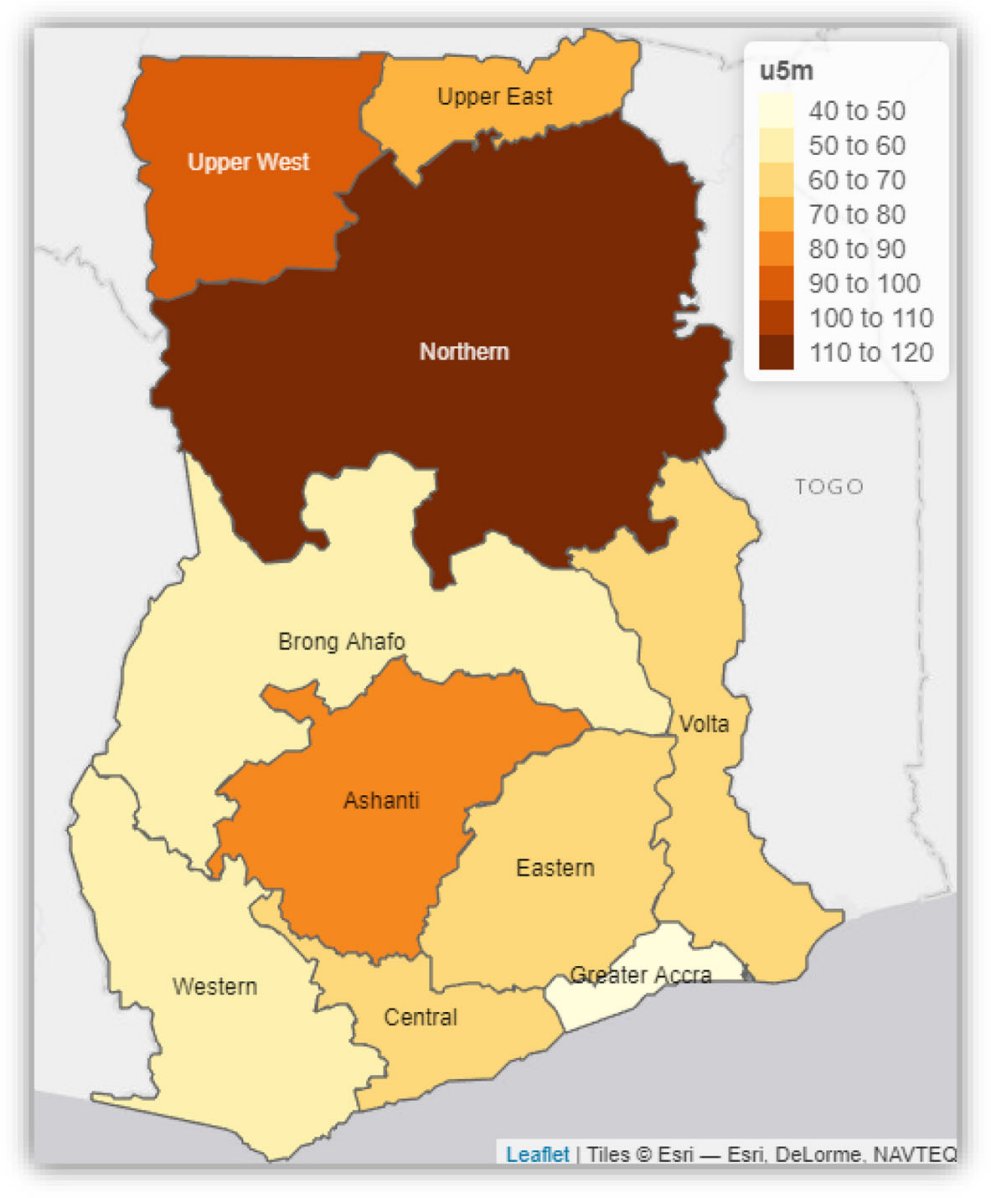
Spatial distribution of crude under-five mortality rates by regions of Ghana using data from the 2014 Ghana Demographic and Health Survey. Source: This map was produced by the author

Figure 2 shows spatial distribution of crude under-five mortality rates by region. The under-five mortality rate in the map is presented as number of under-five deaths per 1000 live births. The Northern region recorded the highest under-five mortality rate of 111 per 1000 live births followed by 92 per 1000 live births in Upper West region. The lowest under-five mortality rate of 47 per 1000 live births was recorded in the Greater Accra region.

## Discussion

The study sets out to develop predictive model and to investigate modifiable risk factors for under-five mortality in Ghana. The study also examined unobserved household level variations in under-five mortality which represents differences in under-five mortality outcome across households that could not be explained by the available risk factors in the model.

The study observed that out of the 5884 children in the data, 289 (4.91%) of them died. Modifiable risk factors associated with under-five mortality were maternal contraceptive use and intention, maternal educational level, number of children under-five in household, total children ever born and number of births in last 5 years. Confounding factors that were significantly associated with under-five mortality were type of birth, sex of child and region of residence.

Of particular interest in this study is the unobserved household level variations in under-five mortality outcome. It is no doubt that the health and general wellbeing of children depend heavily on the households in which they reside. Thus, the opportunities, hazards and resources available to children over their life span are determined by the households [11, 22–24]. No significant unobserved household level variation was found in this study. Thus, the under-five mortality outcome does not vary across households in Ghana after adjusting for the child, household and community level risk factors.

The present study supports previous studies that investigated under-five mortality in developing countries, especially in Sub-Saharan Africa. Children from mothers who do not use and do not intend to use contraceptive, children from mothers with no education, increase in total children ever born, number of births in last 5 years, multiple birth and residing in Central, Volta, Eastern, Ashanti, Brong Ahafo, Northern and Upper West regions are associated with increased odds of under-five mortality whereas been a female child, and increase in number of children under-five in household decreased the odds of under-five mortality [15, 17, 18, 24–27].

Under-five childhood mortality is significantly higher among children belonging to mothers who do not use and do not intend to use contraceptive compared to their counterparts from mothers who use modern contraceptive method. Also, the likelihood of under-five mortality increases with total children ever born and number of births in last 5 years and these findings are consistent with previous studies [15, 18, 25]. All these variables are related to family planning. The use of contraceptive methods may result in adequate birth spacing and could militate against unwanted pregnancies which in turn will reduce health complications such as maternal depletion syndrome and the risk associated with early weaning of the child. Also, increase in total children ever born and number of births in last 5 years could result in lack of care, low birthweight, premature births and heavy drain on the limited household resources as children have to compete for the little resources available for their survival. A previous study also reported that meeting all the contraceptive needs of women could result in 17% reduction in under-five mortality [25, 28, 29].

A reduction in the likelihood of under-five mortality among children of mothers with primary education compared to children of mothers without formal education suggests that improving maternal education will improve the likelihood of child survival outcomes, which is similar to previous findings [14, 26, 27]. This is expected because improvements in educational level of women has been reported to bring several advantages to themselves, their children and the society at large [22, 30]. For example, educated mothers are more likely to develop good health seeking behaviour for themselves and their children, especially utilization of health services, feeding and child care practices which in turn will result in better health outcomes for both mothers and their children. Thus, mother’s education could modify her role in the family and propel her to take critical measures to sound child health outcomes and effective utilization of innovative health services [11, 14, 22, 31, 32].

The likelihood of child survival decreased among children who are products of multiple births compared to their counterparts who were products of singletons. Low birthweight or competition for nutritional intake which occurs more among children who are products of multiple births could be a plausible explanation [11, 22, 25]. This study also showed that being a female child decreased the likelihood of under-five mortality. A plausible explanation could be that a female child is more likely to develop early fetal lung maturity which will be protective of respiratory diseases unlike their male counterparts [18, 26]. The unexpected finding that the risk of under-five mortality decreased with increase in the number of children below 5 years in household could be a reverse causality, which is inconsistent with previous studies [18]. Further research is therefore needed to establish the true direction of the association.

Children residing in Central, Volta, Eastern, Ashanti, Brong Ahafo, Northern and Upper West regions have increased risk of under-five mortality compared to children living in the Western region. The spatial map of crude under-five mortality also revealed regional disparities in crude under-five mortality in Ghana. The rate is highest in the Northern region (111 per 1000 live births) followed by Upper West region (92 per 1000 live births). Greater Accra region recorded the lowest rate (47 per 1000 live births). This study is consistent with previous findings that geographical locations of children influence their health outcomes [14, 15, 18, 26, 27]. In fact, distribution of political power and socioeconomic resources largely influence the health conditions of populations at the local, regional and national levels [33]. In Ghana, there is a persistent regional disparity in the distribution of socioeconomic resources, especially health care delivery, health services and wealth due to lack of political will to implement sound health and economic policies across the regions comprehensively. Also, the regional differences in the under-five mortality could partly be explained by variations in implementation of national health policies and programs coupled with inadequate health services and poor living conditions of households [14, 34–40].

Under-five mortality is an urgent public health problem in Ghana and this study has provided key information for understanding and addressing under-five mortality in the country. The key finding in this study is the crucial role that family planning plays in reducing the risk of under-five mortality. Government and other stakeholders responsible for the health of children and mothers should target meeting the family planning needs of women as part of an overall strategy aimed at reducing the under-five mortality to the level expected in the country. Policies and interventions targeted at improving the level of maternal education could be very beneficial to the survival of children if supported with efforts to improve the general standard of living among households in the country. The strengths of the study are that it is a nationally representative population-based study with good quality data on children, their households and communities in which they reside. The study also has a large sample size drawn randomly nationwide, making it possible to generalise findings to the population of Ghanaian children aged below 5 years and to other similar populations. The multilevel logistic modelling approach also allowed determination of unobserved household level differences in under-five mortality which cannot be determined through single level binary logistic regression. The main limitation of this study is its inability to measure causal effects due to the cross-sectional nature of the study. It is also possible that socioeconomic situations of households at the time of the survey could be different at the time the child died. The retrospective nature of reporting under-five child deaths might result in reporting bias. Furthermore, there are incomplete data on some important variables such as children’s nutritional status, diarrhoea and fever episodes and were not included in the analysis. For example, children who died will not have measurements on their nutritional status as well as on diarrhoea and fever episodes because in the main survey, questions on these variables will not apply to the children who died before the study period.

## Conclusion

Determination of modifiable risk factors associated with under-five mortality is crucial for the identification of which policies and interventions to focus on as well as existing policies and intervention strategies that need to be strengthened in order to reduce under-five child mortality to the level expected. Increased odds of under-five mortality observed for total children ever born, number of births in last 5 years and non-contraceptive use/intention, and the decreased odds of under-five mortality observed for maternal education level in this study revealed the need for family planning and improvements in the level of maternal education as part of the interventions required to reduce the burden of under-five child mortality. Further research on regional disparities in under-five mortality and the discrepancy in the direction of the effect of number of children under-five in households on under-five mortality is warranted to explain these findings.

## Additional files

Additional file 1: **Table S1.** Risk factors for under-five mortality using multilevel logistic regression model (Model 4). (DOCX 22 kb)

Additional file 2: **Table S2.** Goodness of fit test for single level binary logistic (model 3) and multilevel logistic (model 4) regression models. (DOCX 16 kb)

Additional file 3: **Table S3.** Test of multicollinearity using generalised variance inflation factor for logistic regression model (model 3). (DOCX 18 kb)

## Abbreviations

AIC: Akaike Information Criterion
BIC: Bayesian Information Criterion
CHPS: Community-based Health Planning and Services
GDHS: Ghana Demographic and Health Survey
GVIF: Generalised variance-inflation factor (GVIF)
LRT: Likelihood ratio test
MDGs: Millennium Development Goals
ROC: Receiver Operating Characteristic
SDG: Sustainable Development Goal
