# Supplementary material for: Predictive model and determinants of under-five child mortality: evidence from the 2014 Ghana demographic and health survey
Source: BMC Public Health. 2019 Jan 14;19:64. doi: 10.1186/s12889-019-6390-4 (PMC6332681; doi:10.1186/s12889-019-6390-4)
Supplement: Supplementary file 1 — Table S1. Risk factors for under-five mortality using multilevel logistic regression model (Model 4). (DOCX 22 kb) [file 12889_2019_6390_MOESM1_ESM.docx]

Additional file 1: **Table S1** Risk factors for under-five mortality using multilevel logistic regression model (Model 4).

| **Variable aOR (95% CI) Estimate** | |
| --- | --- |
| **Type of birth** |  |
| *Single birth* | 1.00 (Reference) |
| *Multiple birth* | 0.47 (0.30, 0.75)** |
| **Sex of child** |  |
| *Male* | 1.00 (Reference) |
| *Female* | 1.31 (1.00, 1.72)* |
| **Respondent's current age** | 1.03 (0.99, 1.06) |
| **Highest education level** |  |
| *No education* | 1.00 (Reference) |
| *Primary* | 1.45 (0.96, 2.19) |
| *Secondary* | 1.22 (0.82, 1.81) |
| *Higher* | 0.95 (0.43, 2.07) |
| **Religion** |  |
| *Islam* | 1.00 (Reference) |
| *Christian* | 1.06 (0.73, 1.54) |
| *Traditionalist/spiritualist* | 1.13 (0.52, 2.48) |
| *No religion* | 0.89 (0.45, 1.76) |
| **Number of children <5 years** | 7.20 (5.80, 8.93)*** |
| **Wealth index** |  |
| *Rich* | 1.00 (Reference) |
| *Poor* | 1.19 (0.72, 1.96) |
| *Average* | 1.15 (0.72, 1.82) |
| **Total children ever born** | 0.81 (0.73, 0.90)*** |
| **Births in last five years** | 0.16 (0.13, 0.21)*** |
| **Contraceptive use/ intention** |  |
| *Using modern method* | 1.00 (Reference) |
| *Using traditional method* | 2.20 (0.52, 9.39) |
| *Non-user - intends to use later* | 0.76 (0.51, 1.13) |
| *Does not intend to use* | 0.56 (0.37, 0.82)** |
| **Covered by health insurance** |  |
| *No* | 1.00 (Reference) |
| *Yes* | 0.99 (0.73, 1.34) |
| **Place of residence** |  |
| *Urban* | 1.00 (Reference) |
| *Rural* | 1.03 (0.72, 1.49) |
| **Region** |  |
| *Western* | 1.00 (Reference) |
| *Central* | 0.43 (0.22, 0.85)* |
| *Greater Accra* | 0.90 (0.38, 2.13) |
| *Volta* | 0.39 (0.18, 0.83)* |
| *Eastern* | 0.33 (0.17, 0.65)** |
| *Ashanti* | 0.46 (0.23, 0.90)* |
| *Brong Ahafo* | 0.48 (0.23, 0.97)* |
| *Northern* | 0.26 (0.13, 0.51)*** |
| *Upper east* | 0.48 (0.22, 1.04) |
| *Upper west* | 0.38 (0.19, 0.77)** |
| **Household level variance** | 2.8e-13 |

aOR: Adjusted odds ratio. CI: Confidence interval. *: p-value <0.05. **: p-value <0.01. ***: p-value <0.001
