# Supplementary material for: Predictive model and determinants of under-five child mortality: evidence from the 2014 Ghana demographic and health survey
Source: BMC Public Health. 2019 Jan 14;19:64. doi: 10.1186/s12889-019-6390-4 (PMC6332681; doi:10.1186/s12889-019-6390-4)
Supplement: Supplementary file 2 — Table S2. Goodness of fit test for single level binary logistic (model 3) and multilevel logistic (model 4) regression models. (DOCX 16 kb) [file 12889_2019_6390_MOESM2_ESM.docx]

Additional file 2: **Table S2** Goodness of fit test for single level binary logistic (model 3) and multilevel logistic (model 4) regression models

| Model Degrees of freedom AIC BIC Deviance P-value |
| --- |
| Model 3 29 1762.6 1956.3 1704.6  Model 4 30 1764.6 1965.0 1704.6 1.0 |

AIC=Akaike Information Criterion, BIC=Bayesian Information Criterion.
