# Supplementary material for: Predictive model and determinants of under-five child mortality: evidence from the 2014 Ghana demographic and health survey
Source: BMC Public Health. 2019 Jan 14;19:64. doi: 10.1186/s12889-019-6390-4 (PMC6332681; doi:10.1186/s12889-019-6390-4)
Supplement: Supplementary file 3 — Table S3. Test of multicollinearity using generalised variance inflation factor for logistic regression model (model 3). (DOCX 18 kb) [file 12889_2019_6390_MOESM3_ESM.docx]

Additional file 3: **Table S3** Test of multicollinearity using generalised variance inflation factor for logistic regression model (model 3)

| **Variable GVIF Degrees of freedom (DF) GVIF^(1/2*DF)** | | | |
| --- | --- | --- | --- |
| Type of birth | 1.17 | 1.00 | 1.08 |
| Sex of child | 1.02 | 1.00 | 1.01 |
| Respondent's current age | 2.87 | 1.00 | 1.69 |
| Highest education level | 2.13 | 3.00 | 1.13 |
| Religion | 1.62 | 3.00 | 1.08 |
| Number of children <5 years | 1.82 | 1.00 | 1.35 |
| Wealth index | 2.76 | 2.00 | 1.29 |
| Total children ever born | 3.57 | 1.00 | 1.89 |
| Births in last five years | 2.07 | 1.00 | 1.44 |
| Contraceptive use/ intention | 1.16 | 3.00 | 1.02 |
| Covered by health insurance | 1.10 | 1.00 | 1.05 |
| Place of residence | 1.85 | 1.00 | 1.36 |
| Region | 2.74 | 9.00 | 1.06 |
|  | | | |

AIC=Akaike Information Criterion, BIC=Bayesian Information Criterion.
